# Case Report: Disappearance of Coronary Anastomotic Aneurysm by Steroid Therapy in Takayasu Arteritis: Pseudorepair of Pseudoaneurysm?

**DOI:** 10.3389/fcvm.2021.683216

**Published:** 2021-06-04

**Authors:** Shuichi Naraoka, Hiroki Uchiyama, Toshiyuki Yano, Takuma Mikami, Ryo Harada, Yosuke Kuroda, Yuki Toda, Atsuko Muranaka, Taro Sugawara, Tadashi Hasegawa, Tetsuji Miura, Nobuyoshi Kawaharada

**Affiliations:** ^1^Department of Cardiovascular Surgery, Sapporo, Japan; ^2^Department of Cardiovascular, Renal and Metabolic Medicine, Sapporo, Japan; ^3^Department of Surgical Pathology, Sapporo Medical University School of Medicine, Sapporo, Japan

**Keywords:** takayasu arteritis, aneurysm, aortic regurgitation, ruptured sinus of valsalva, steroid

## Abstract

**Background:** Takayasu arteritis (TA) is a large vessel vasculitis of unknown etiology characterized by chronic inflammatory changes of the aorta and its major branches. Complications such as anastomotic aneurysm and valve detachment have been reported in active TA patients who received aortic valve replacement and graft replacement of aorta.

**Case Summary:**A 61-year-old man with a history of emergency aortic valve replacement and patch closure of the noncoronary sinus of Valsalva due to acute heart failure induced by acute aortic regurgitation and ruptured sinus of Valsalva 4 years ago was referred for exertional dyspnea. Dilatation of the sinus of Valsalva together with protrusion of the right sinus of Valsalva and ostial stenosis of the left coronary artery were newly found by computed tomography (CT). A Bentall operation with concomitant coronary artery bypass grafting was successfully performed with a composite graft. Diagnosis of TA was made on the basis of histological analyses of the resected sinus of Valsalva, though other arterial manifestations were not detected by ^18^F-fluorodeoxyglucose (^18^F-FDG) positron emission tomography/computed tomography. Three months later, a follow-up study revealed left coronary ostial pseudoaneurysm at the anastomotic site together with strong ^18^F-FDG uptake, leading to commencement of steroid therapy. Eight months later, disappearance of left coronary ostial pseudoaneurysm was found by a follow-up CT scan.

**Conclusion:** This is a rare TA case in whom spontaneous resolution of coronary anastomotic aneurysm by steroid therapy was found without reconstructive surgery.

## Introduction

Takayasu arteritis (TA) is a large vessel vasculitis of unknown etiology characterized by chronic inflammatory changes of the aorta and its major branches (1, 2). Treatment of TA consists of two strategies: immunosuppressive therapy and management of vascular diseases including surgical/interventional procedures.

## Case Presentation

**Figure 1:**
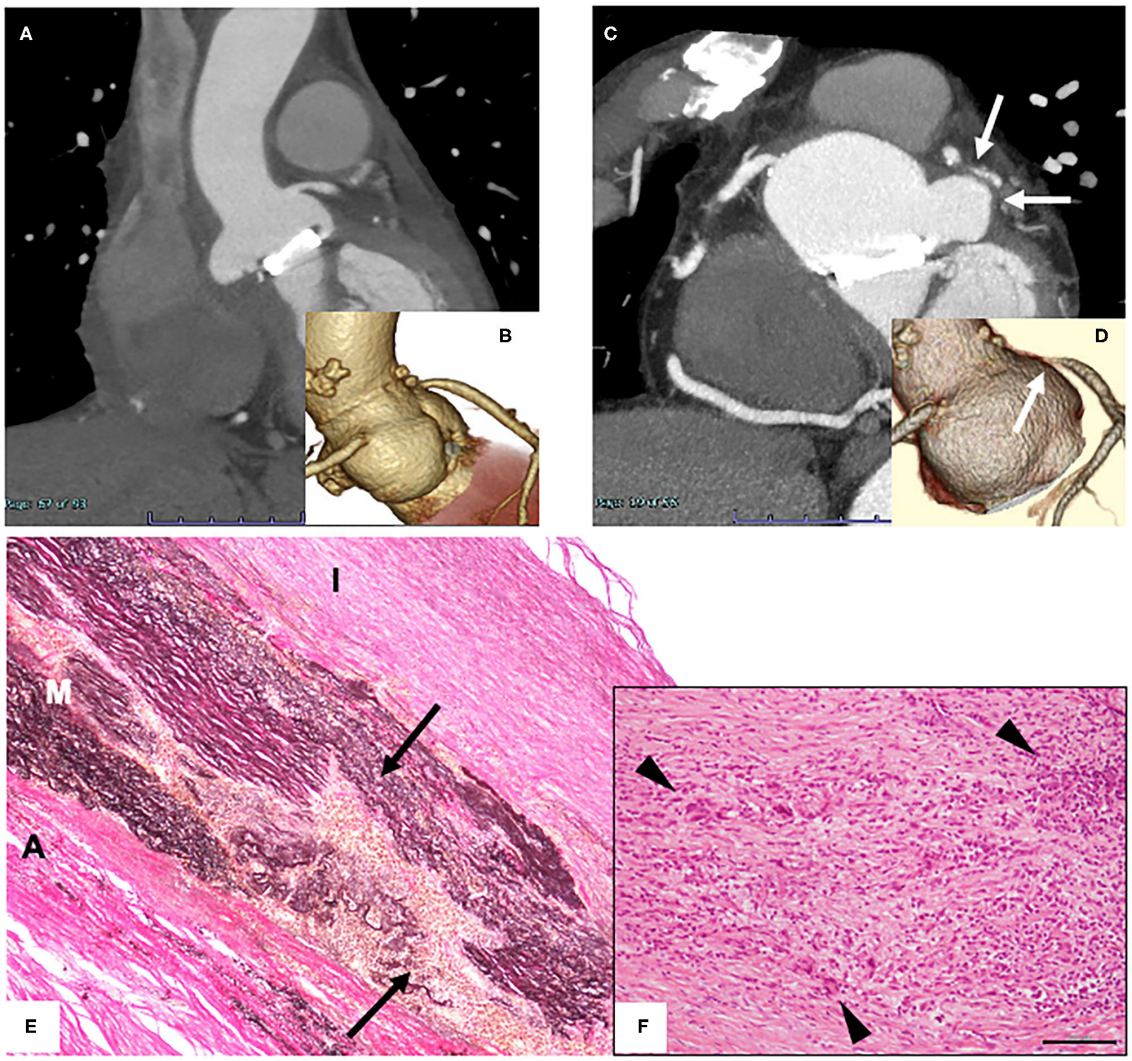
**(A,C)** Contrast-enhanced computed tomography images 4 years ago **(A)** and on admission **(C)**. **(B,D)** 3D coronary angiography 4 years ago **(B)** and on admission **(D)**. **(E,F)** Histological findings of surgically resected tissue of the sinus of Valsalva. Images of Elastica von Gieson staining [**(E)**; original magnification 100x] and hematoxylin and eosin staining [**(F)**; original magnification 400x; scale bar, 100 mm] are shown. A, adventitia; M, media; I, intima. Arrows indicate the site with disruption of elastic fibers in the medial layer. Arrow heads indicate giant cells.

A 61-year-old man with a history of emergency aortic valve replacement (AVR) and patch closure of the noncoronary sinus of Valsalva due to acute heart failure induced by acute aortic regurgitation and ruptured sinus of Valsalva 4 years ago was referred for exertional dyspnea. The results of histological examination of resected aortic valves after AVR were unremarkable. Dilatation of the sinus of Valsalva together with protrusion of the right sinus of Valsalva, which had not been observed 4 years ago, were found by computed tomography (CT, Figures 1A–D). In addition, the CT images revealed severe ostial stenosis of the left coronary artery and mild ostial stenosis of the right coronary artery (Figures 1A–D). Laboratory data revealed an elevated C-reactive protein (CRP) level of 1.06 mg/dL together with mild anemia and high level of fibrinogen (516 mg/dL). There was a trend for neutrophil left shift (74.2%) without enhanced white blood cell count. Urinalysis and liver/renal function tests were normal. A Bentall operation with concomitant coronary artery bypass grafting was successfully performed with a composite graft (28-mm Gelweave graft, Vascutek). In histological analyses of the resected sinus of Valsalva, massive infiltration of lymphocytes, histiocytes, and giant cells mainly in the media and adventitia with destruction of the media was found (Figures 1E,F). In addition, the adventitia was massively expanded. These histological findings of aorta were consistent with the characteristics of TA. The patient carried the HLA-B52 allele, which is frequently found in Japanese TA cases, whereas the patient did not carry the HLA-DRB1^*^04, which have been shown to be associated with development of giant cell arteritis (GCA). A treponema pallidum hemagglutination assay yielded a negative result and serum IgG4 level was not elevated, leading to the exclusion of syphilitic aortitis and IgG4-related disease. Considering that the patient did not have history of symptoms suggesting temporal artery involvement and polymyalgia rheumatica, characteristics of GCA, a diagnosis of TA was made. Although other arterial manifestations were not detected by ^18^F-fluorodeoxyglucose (^18^F-FDG) positron emission tomography/computed tomography, commencement of steroid therapy was planned after healing of sites of median sternotomy to avoid surgical wound dehiscence.

**Figure 2:**
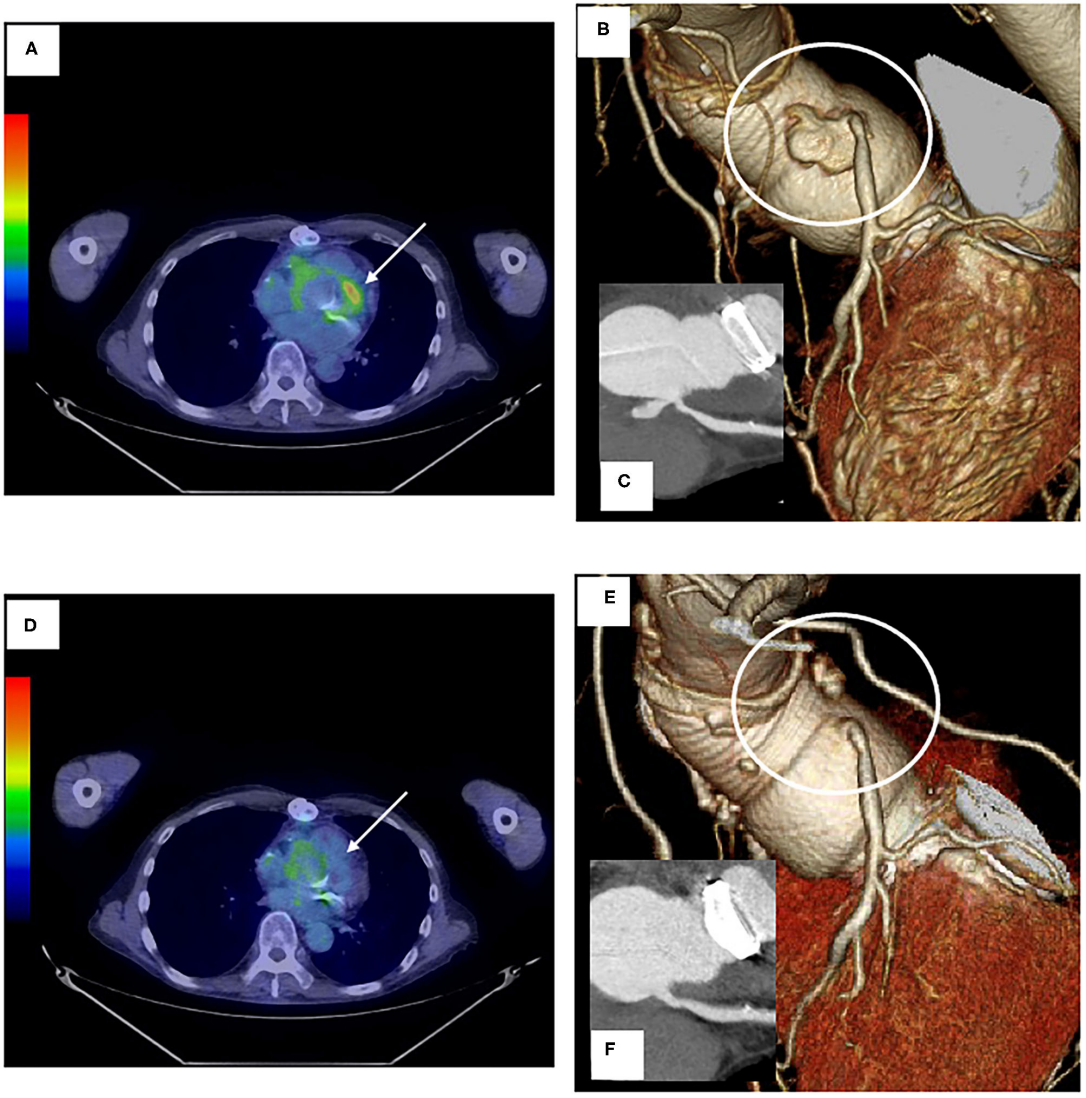
**(A,D)** Images of ^18^F-fluorodeoxyglucose (^18^F-FDG) positron emission tomography/computed tomography before **(A)** and after **(D)** steroid therapy. Arrows indicate the anastomotic site with left coronary artery aneurysm. **(B,C,E,F)** 3D coronary angiography and contrast-enhanced CT images before **(B,C)** and after steroid administration **(E,F)**.

**Figure 3:**
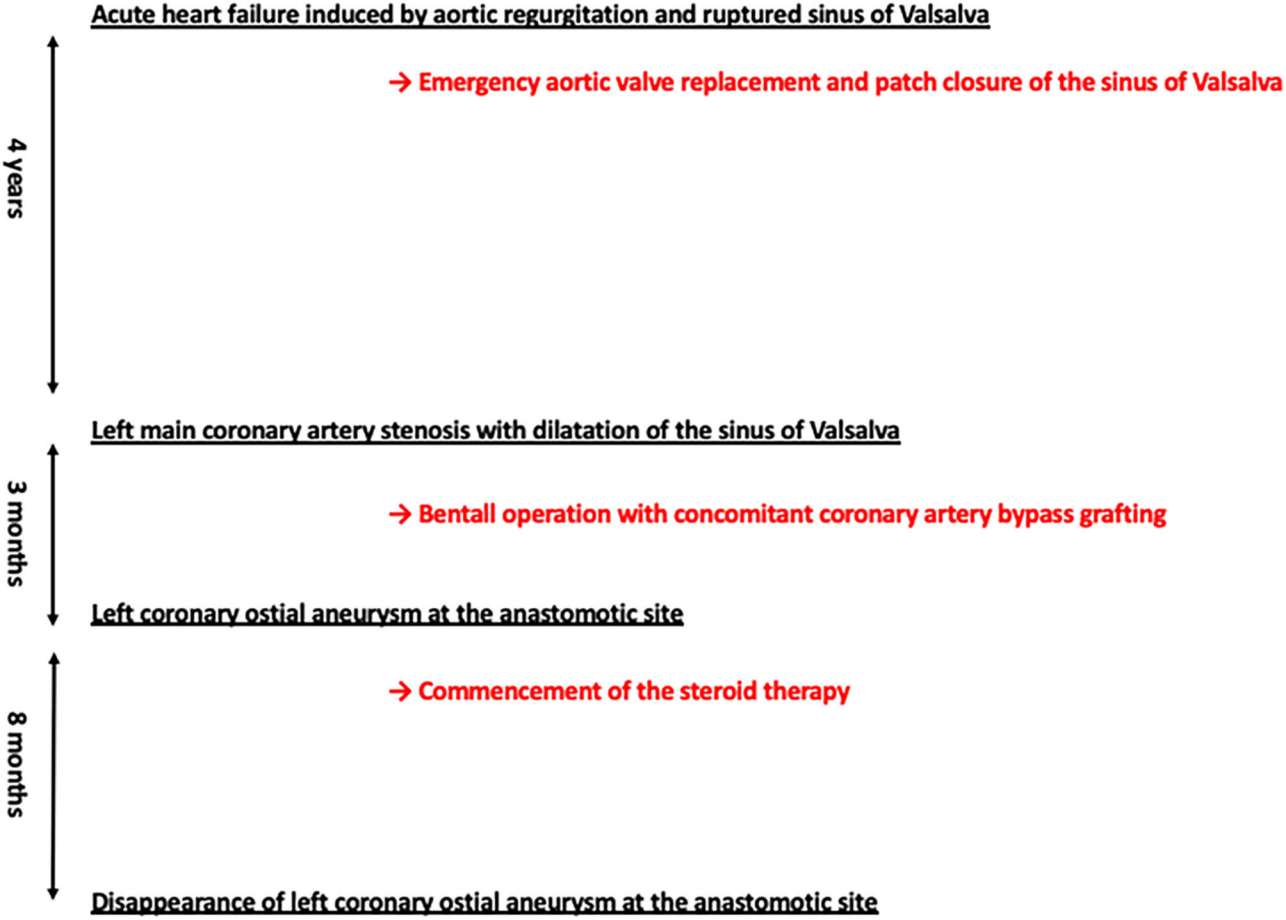
Clinical courses of this case.

Approximately 3 months later, a follow-up study revealed a left coronary ostial aneurysm at the anastomotic site together with strong ^18^F-FDG uptake (Figures 2A–C), which was not detectable by transthoracic echocardiography. Repeated blood cultures yielded negative results. There were two concerns for surgical correction of coronary aneurysm: (1) the patient had already received repeated thoracotomies including a thoracotomy performed 3 months ago and (2) risk of repeated complications at the anastomotic sites would be high since he had active TA. For this reason, treatment with 40 mg of prednisolone per day together with medical therapy to relieve myocardial ischemia was commenced with careful follow-up of the affected site prior to surgical correction. Disappearance of strong ^18^F-FDG uptake was found a month after the commencement of steroid therapy (Figure 2D). Eight months later, the prednisolone dosage was reduced to 10 mg per day without re-elevation of CRP. A CT scan showed disappearance of the left coronary ostial pseudoaneurysm (Figures 2E,F). Clinical courses of this case were summarized in Figure 3.

## Discussion

There are some clues to differentiate two diseases, though it remains unclear whether GCA and TA, predominant causes of large vessel vasculitis, are different clinical entities (3–6). The age of onset is usually younger in patients with TA (<40 years) than in patients with GCA (>50 years). GCA is usually associated with polymyalgia rheumatica, resulting in repeated episodes of fever, and is also associated with involvement of temporal artery, leading to a higher prevalence of headache (3–6). There are differences in genetic backgrounds between two diseases: GCA is associated with genes in the HLA class II, while TA is associated with those in the HLA class I including the HLA-B52 gene (3–6). Although GCA and TA share similar pathological features, GCA is usually associated with severe inflammation in the inner and middle layer of the media, whereas TA is associated with the outer layer of the media to the adventitia and a marked thickening of adventitia (3–7). In addition, copious infiltration of giant cells and well-formed granulomas are usually observed in large vessels of GCA (7). Clinical and pathological characteristics of our case other than the age of onset suggest a diagnosis of TA. However, a careful follow-up is needed to detect the development of GCA-related manifestations to support a diagnosis of TA.

Complications after AVR such as anastomotic aneurysm and valve detachment have been reported in patients with TA (8). A previous study by Miyata et al. showed that the cumulative incidence of anastomotic aneurysm during a 20-year follow-up period was 12.0% (9). Importantly, the presence of aneurysmal lesions before surgery, but not the presence of systemic inflammation or use of a steroid, was shown to be associated with the development of an anastomotic aneurysm after surgery (9). However, the association of development of an anastomotic aneurysm with histologic inflammation was not extensively analyzed in that study. On the other hand, results of a previous study by Matsuura et al. showed that active inflammation of the resected aortic wall was a risk factor of valve/graft detachment in TA patients who received surgical treatment (AVR or composite graft repair) for aortic regurgitation (9). In cases such as this case in which emergency surgery is performed due to acute heart failure and in which arterial manifestation of TA is limited to the sinus of Valsalva, it will not be easy to predict the presence of active inflammation due to TA at the time of the first operation. Nevertheless, thorough imaging studies and careful monitoring by serological inflammatory markers should be performed to detect complications after AVR or Bentall operation if there are clinical suspicions of TA such as episodes of acute valvular dysfunction and a ruptured sinus of Valsalva.

There are two pathologically different forms of aneurysm: true aneurysm and pseudoaneurysm due to dissection or rupture of an arterial wall (10), both of which are associated with TA. Although it was not easy to perform intravascular ultrasonography to obtain information on the arterial wall structure in our case with active TA, rapid development of an aneurysm at the anastomotic site in addition to findings showing active inflammation, i.e., histological findings of the sinus of Valsalva and strong ^18^F-FDG uptake, indicated pseudoaneurysmal formation rather than true aneurysmal formation that develops during natural history of active TA since development of anastomotic pseudoaneurysm has been shown to be not an unusual finding in TA patients who received AVR by shown in previous studies (8, 9). A plausible explanation of the favorable effect of steroid therapy on pseudoaneurysm is that the ruptured vessel wall was encapsulated by fibrotic tissue formed by steroid therapy and the encapsulated lumen was completely sealed with the thrombus, leading to radiographic disappearance of the aneurysm, i.e., pseudorepair of pseudoaneurysm. Although prompt surgical/interventional therapy is a first-line therapy for a pseudoaneurysm to avoid serious complications such as rupture (11, 12), preceding steroid therapy may be an alternative approach in active TA cases with an anastomotic coronary pseudoaneurysm. However, a careful follow-up of affected site and other large vessels is needed since results of a retrospective cohort study by Tajima et al. revealed that long-term oral steroid therapy may be a risk factor for expansion of abdominal aortic aneurysm (13), though it remain unclear whether this is the case with vessels with pseudoaneurysm.

## Data Availability Statement

The original contributions presented in the study are included in the article/supplementary material, further inquiries can be directed to the corresponding author.

## Ethics Statement

Written informed consent was obtained from the individuals for the publication of any potentially identifiable images or data included in this article.

## Author Contributions

All authors listed have made a substantial, direct and intellectual contribution to the work, and approved it for publication.

## Conflict of Interest

The authors declare that the research was conducted in the absence of any commercial or financial relationships that could be construed as a potential conflict of interest.
